# Multiphase Reconstruction of Heterogeneous Materials Using Machine Learning and Quality of Connection Function

**DOI:** 10.3390/ma17133049

**Published:** 2024-06-21

**Authors:** Pouria Hamidpour, Alireza Araee, Majid Baniassadi, Hamid Garmestani

**Affiliations:** 1 School of Mechanical Engineering, College of Engineering, University of Tehran, Tehran 14155-6619, Iran; pouriahamidpour@ut.ac.ir (P.H.); alaraee@ut.ac.ir (A.A.); 2 University of Strasbourg, CNRS, ICube UMR 7357, 67081 Strasbourg, France; 3 School of Materials Science and Engineering, Georgia Institute of Technology, 771 Ferst Drive NW, Atlanta, GA 30332, USA

**Keywords:** 3D microstructure reconstruction, transfer learning, convolutional occupancy networks, serial-section stitching, point cloud data, multi-phase heterogeneous materials, quality of connection function, statistical function

## Abstract

Establishing accurate structure–property linkages and precise phase volume accuracy in 3D microstructure reconstruction of materials remains challenging, particularly with limited samples. This paper presents an optimized method for reconstructing 3D microstructures of various materials, including isotropic and anisotropic types with two and three phases, using convolutional occupancy networks and point clouds from inner layers of the microstructure. The method emphasizes precise phase representation and compatibility with point cloud data. A stage within the Quality of Connection Function (QCF) repetition loop optimizes the weights of the convolutional occupancy networks model to minimize error between the microstructure’s statistical properties and the reconstructive model. This model successfully reconstructs 3D representations from initial 2D serial images. Comparisons with screened Poisson surface reconstruction and local implicit grid methods demonstrate the model’s efficacy. The developed model proves suitable for high-quality 3D microstructure reconstruction, aiding in structure–property linkages and finite element analysis.

## 1. Introduction

The investigation of microstructures in three dimensions (3D) is driven by numerous motivations. Despite advancements, significant gaps persist in precise reconstruction of the structure of the materials and in comprehending the behavior of materials. These gaps highlight the ongoing need for comprehensive research in this field. To motivate this research, it is necessary to note that most investigations need to consider the precise inherently three-dimensional microstructure of the materials [1]. Most simulation problems need an accurate definition of the initial condition, which includes a description of the internal structure of the materials for a problem. Therefore, achieving this structure of the materials is very important [2,3,4].

Over the past year, various initiatives have been undertaken to attain the three-dimensional structure of materials [5,6,7,8]. The initial efforts focused on achieving the materials’ structure through practical methodologies including microtomography and stochastic reconstruction of the microstructure [9,10,11]. Practical methods prove to be both costly and time-consuming [12]. Moreover, conventional stochastic reconstruction methods are prone to relatively high inaccuracies and their iterative processes impose a significant computational burden [13,14]. In recent years, to achieve 3D microstructures of materials, different deep learning and transfer learning approaches were developed [15,16,17]. These methods have proven to deliver superior results compared to conventional stochastic methods, showing an enhanced ability to reconstruct the intricate geometries present in the structures of various materials [18,19]. Li et al. [20] employed a transfer learning approach for the 3D reconstruction of materials. They introduced a process involving encoder–decoder architecture and feature-matching optimization through a deep convolutional network, specifically designed for the 3D reconstruction of diverse materials.

Bostanabad [21] pioneered a transfer learning technique for the 3D reconstruction of materials based on 2D images of microstructures. Employing extrapolation-based reconstruction, the method proves effective across a diverse range of microstructures encompassing alloys, porous media, and polycrystalline materials. In this developed model, a random 3D image undergoes iterative refinement to align its microstructural features with those of an exemplar. The approach utilizes a trained two-dimensional VGG model, wherein only orthogonal images of blocks related to internal voxels are inputted into the model as two-dimensional images, and subsequently compared with the initial single two-dimensional image.

Xu et al. [22] introduced a method that predicts the mechanical properties of a two-phase composite using a reduced dataset through transfer learning. Their approach involves presenting an analytical framework for composite microstructure characteristics, which is utilized to obtain sample labels. This facilitates the creation of a sufficient dataset, employed for the pre-training of the initial convolutional neural network (CNN) in the source domain during the transfer learning process.

One primary limitation of the proposed methods lies in the process of reconstructing a three-dimensional structure, which involves synthesizing two-dimensional images for small blocks within the microstructure, showcasing similar characteristics to the input images. These images serve the dual purpose of representing both the outer shell of the microstructure and the shells of the inner blocks. Additionally, these methods exclusively utilize a single input image. Furthermore, in the mentioned transfer learning approaches, the only criterion for reconstruction is the presence of statistical features resembling the initial input image. Consequently, this implies that their reconstruction is more similar to synthesis, lacking consideration for the matching of depth layers.

To address the limitations inherent in these methods and achieve a more precise 3D microstructure representation of materials with different characteristics, the authors developed a transfer learning method. This method involves an interpolation synthesis of the depth cuts of the materials followed by the subsequent 3D reconstruction of the materials using convolutional occupancy networks.

The proposed method requires that the depth sections of the reconstructed three-dimensional microstructure align precisely with the original depth two-dimensional microstructures. Additionally, this innovative approach represents the first instance of utilizing a point cloud input imported from the initial images for the 3D reconstruction process. Notably, the novel method not only produces three-dimensional reconstructions with statistical characteristics similar to the corresponding layers but also places a significant emphasis on achieving appearance similarity and reconstruction accuracy at each layer through the employed reconstruction model.

However, the main purpose of this study is incorporating an enhanced optimization process by introducing the Quality of the Connection Function (QCF) into the model [23]. The aim is to achieve heightened accuracy in phase volume within the 3D microstructure reconstruction of materials through the application of transfer learning techniques. A transfer learning model is established utilizing the convolutional occupancy networks concept to generate three-dimensional microstructure reconstructions of the isotropic and anisotropic two- and three-phase materials. This is achieved through point clouds extracted from the inner layers of a material’s microstructure. Within this framework, emphasis is placed not only on accurately portraying the phases of the microstructure but also on ensuring their compatibility with the point cloud data. The optimization process is designed to minimize the error between the statistical properties of the microstructure and the reconstructed model. The model’s initial phase incorporates transfer learning from a pre-trained model, while the subsequent phase utilizes an optimization approach to establish the input model for the multiresolution iso-surface extraction model [24]. To initiate, the model convolutional occupancy networks are trained on a comprehensive database and used for 3D reconstruction of the structure of the aforementioned materials. To demonstrate the model’s capabilities and validate its performance, the same procedure was employed for the 3D reconstruction of the same materials’ structures using references [25,26]. Among these methods, local implicit grid representations (LIG) for 3D scenes are notable, specifically designed for reconstructing 3D objects from partial or noisy data. LIG uses localized fitting of implicit surfaces, allowing for detailed and accurate reconstruction of complex surfaces. Additionally, Screened Poisson Surface Reconstruction (SPSR) was also employed as another method in the reconstruction process. SPSR involves creating surfaces from point clouds by solving the Poisson equation, which can naturally fill gaps in data and produce smooth surfaces. The results are compared with our model both visually and statistically using correlation functions.

## 2. Materials and Methods

### 2.1. Initial Input

The database was established through our in-house software, which features a Monte Carlo approach designed for generating diverse virtual 2D microstructures of two- and three-phase isotropic and anisotropic materials. Utilizing our previously developed realization technique, we generated a total of 100 microstructures for each state using this software.

Our realization approach encompasses three primary steps: generation, distribution, and growth of cells. Within this technique, nucleation, grain growth, and the initial distribution of seeds are crucial factors governing the state of heterogeneity, as well as the size and distribution of phases throughout the realization process. Specifically, nucleation and grain growth play pivotal roles in a virtual realization, influencing the extent of heterogeneity.

The realization process involves cells (alternatively referred to as grains or particles), representing the initial geometries assigned to each phase before the growth step. In each realization, multiple initial cells are placed at random nucleation points for different phases. After the assignment of initial cell geometries, the growth of cells commences through a cellular automaton algorithm.

During each iteration of the growth step, the state of each cell undergoes updates based on the states of neighboring cells, following specific growth rules, also known as transition functions. In this study, the Neumann-type neighborhood relationship is employed, where each cell considers six adjacent cells on its top, bottom, right, left, front, and back for its subsequent growth state. The update rules can be deterministic or stochastic and applied synchronously or asynchronously. The growth of cells continues until they meet each other, filling the grid with three phases. It is important to note that a hard-core (non-penetrating) condition is consistently maintained throughout the initial distribution and growth of the cells. Further details of the realization procedure can be found in our previous articles [1,27].

This study focuses on amorphous materials, utilizing their inherent structural randomness to develop and validate our 3D microstructure reconstruction method. For isotropic two-phase materials, one phase constitutes 47% of the volume, while for anisotropic two-phase materials, it is 53%. In three-phase isotropic materials, one phase accounts for 63%, and another phase 17%, whereas in anisotropic three-phase materials, one phase is 42% and another phase 23%, with the remaining volume comprising the third phase.

**Figure 1:**
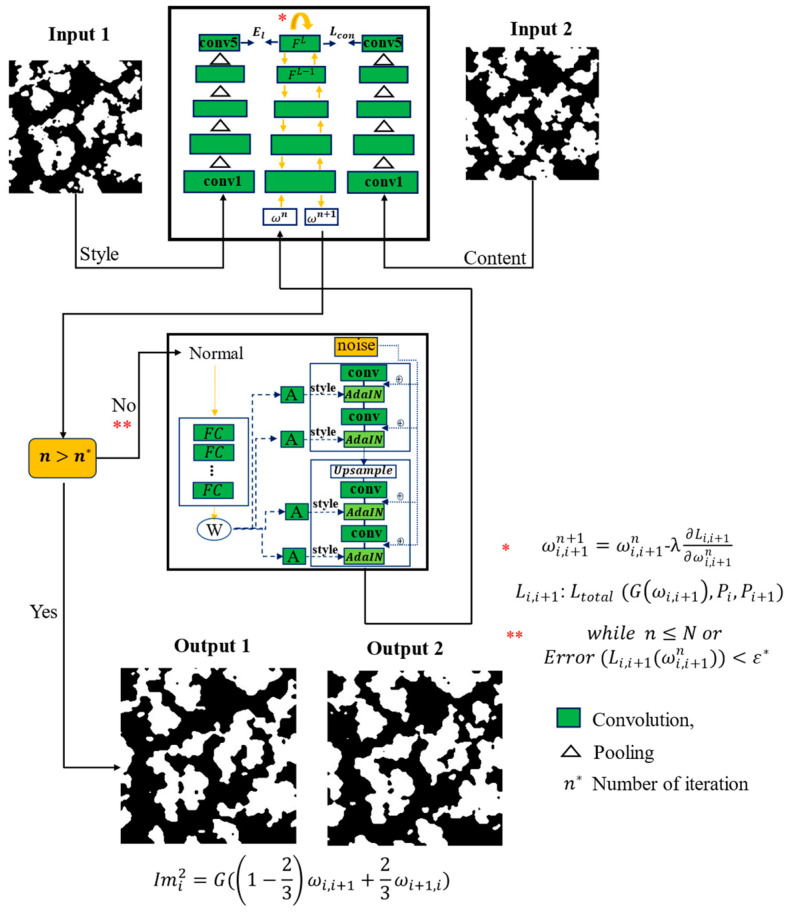
The model used for interpolation synthesis of the 2D images of the microstructure from initial inputs.

Initially, 100 2D images of the microstructure are used and additionally, 198 other 2D images of the microstructure of the materials are synthesized using transfer learning based on StyleGAN network interpolation and the two-way Gatys model. This approach involves transferring the pre-trained model and dividing the process into two sections: content representation and style representation. In the Gatys method, only the convolution network portion of the transferred model is utilized. This model uses two input images and reconstructs two output images between these images. The schematic of the model used for interpolating and synthesizing the 2D images of the microstructure of materials is depicted in Figure 1. StyleGAN employs an eight-layer multilayer perceptron (MLP) model to map the initial code’s latent vector to latent space. This procedure is added to each convolution layer of the generator model using adaptive instance normalization (AdalN). AdaIN aligns the mean and variance of the content features to match those of the style features, making it a normalization technique. We utilize the two-way Gatys model to optimize the latent space to define the error function. This approach involves transferring the pre-training model and dividing the process into two sections: content representation and style representation. In the Gatys method, only the convolution network portion of the transferred model is utilized. In this model, it is assumed that there are $N_{l}$ features in the convolutional layer l, each with the size of $M_{l}$ one-dimensional index. Therefore, $F^{l}\in r^{N_{l}\times M_{l}}$ is defined as the matrix corresponding to the l-th layer, where $F_{i,j}^{l}$ corresponds to the i-th filter and j-th coordinates. To interpolate between two images $P_{i}$ and $P_{i+1}$ at depths i and i+1 of I, it is assumed that ω is a latent code or input Gaussian random image to the styleGAN model; the output of the G(ω) generator is considered as the input image to optimize the latent space in the styleGAN model.

(1) $$L_{i,i+1}:L_{total}\left(G\left(\omega \right),P_{i}\right)=\alpha L_{content}\left(G\left(\omega \right),P_{i}\right)+\beta L_{style}\left(G\left(\omega \right),P_{i}\right)+\gamma \;∥G\left(\omega \right)-P_{i}{∥}_{g}^{2}$$

Then, using the conjugate gradient method, the optimization of ω space will be as follows:

(2) $$\omega_{i,i+1}^{n+1}=\omega_{i,i+1}^{n}-\lambda \frac{\partial L_{i,i+1}}{\partial \omega_{i,i+1}^{n}}\;\;\;\;while\;n\leq N\;or\;Error\;(L_{i,i+1}(\omega_{i,i+1}^{n}))<\varepsilon^{*}$$

In Relation (2), N defines the number of repetitions or the error limit of the loss function and $\varepsilon^{*}$ will be the stopping criterion. To interpolate and synthesize the 2D microstructure between the image of $P_{i}$ and $P_{i+1}$, latent space of $\omega_{i,i+1}$ and $\omega_{i+1,i}$ is calculated using the optimization procedure above.

(3) $${Im}_{i}^{t}=G(\left(1-\frac{t}{S}\right)\omega_{i,i+1}+\frac{t}{S}\omega_{i+1,i})$$

### 2.2. Representation of Optimized Transfer Learning Method for 3D Reconstruction of the Structure

**Figure 2:**
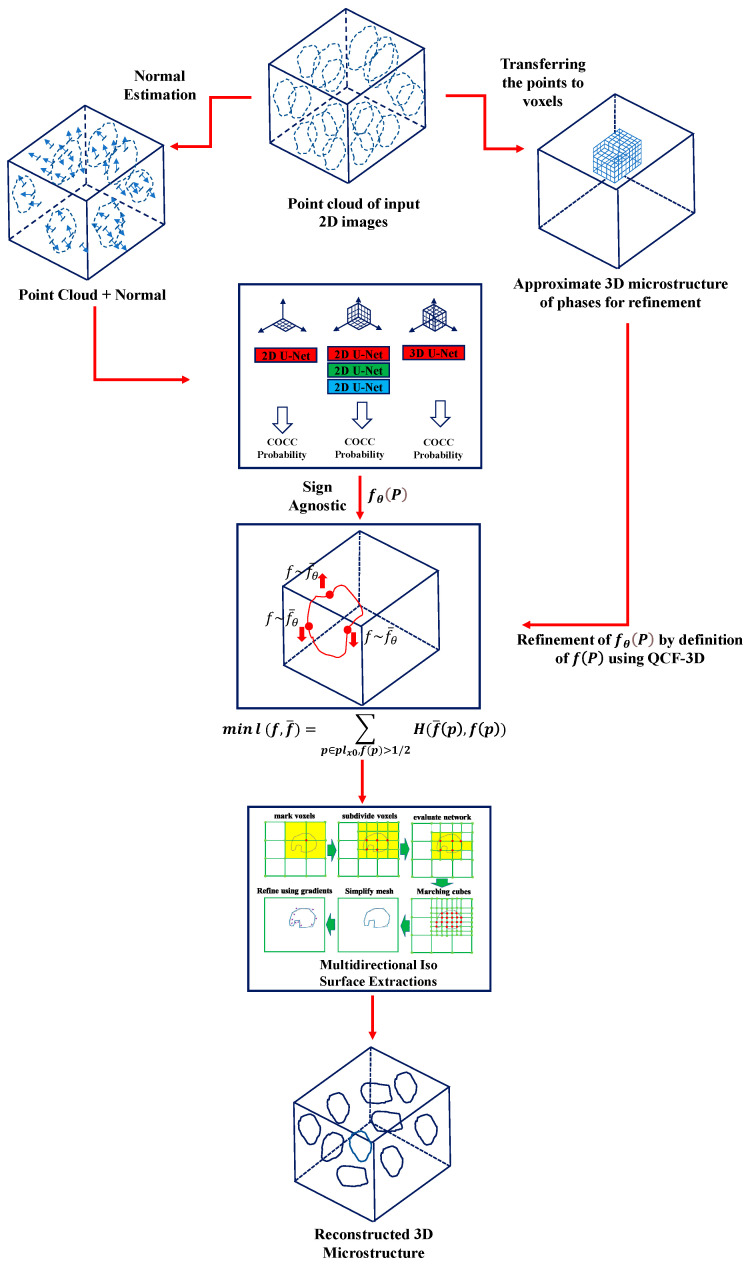
Schematic representation of the developed optimized model for 3D reconstruction of the structure.

As mentioned, transfer learning was employed to synthesize microstructures in a 3D state. Moreover, an optimization approach has been integrated into the model. This optimization step is aimed at ensuring that the statistical compatibility of cross-sections in the three-dimensional mode closely matches the original structure before generating the phase shells. This is essential to prevent issues like non-connectivity or phase breakage during the construction of intricate and irregular microstructures. To establish the optimization stage, the output model’s probability of occupancy is employed within the optimization framework, bridging the gap between the cuts of the output model and those of the input point clouds. This optimization process enhances the accuracy of the mean squared error (MSE) by optimizing the occupancy probability. The schematic representation of the model is presented in Figure 2.

In order to enhance the accuracy of the model, the initial point cloud model is first transferred into the form of voxels to obtain the initial three-dimensional model needed for comparison. This research introduces the three-dimensional QCF calculated in volume for comparing the model in volume form with the aforementioned initial model. In other words, the reconstruction of the microstructure is achieved through synthesis by incorporating an optimization form based on QCF. This addition enhances the value of the reconstruction in terms of its structural accuracy.

Consequently, during the synthesis process, the model’s visual output will be evaluated in addition to the statistical criteria, comparing it with other methods.

Two-dimensional images of microstructure layers serve as the foundation for generating a point cloud that constitutes the input for this optimization task.

At the first stage, the point cloud needs to be converted into voxels. To achieve this, we consider the point cloud as consisting of points on the edges of phases, visible in two-dimensional material sections. Consequently, the data are generated by layering the model in a way that ensures small distances between the layers.

To create voxel-shaped points, we expand the dimensions of points into cubes that encompass a geometric space. These voxel-shaped points are generated from the cubes defined in the form of $\left[i,j,k,i+1,j+1,k+1\right]$, as defined in Relation (4).

(4) $$VX=\underset{i,j,k\in \left[o.N\right]}{⋂}\{\left[\left(i,j,k\right).(i+1,j+1,k+1)\right]\;\exists p\in PC:p\;\in \left[\left(i,j,k\right).(i+1,j+1,k+1)\right]\}$$

The subsequent stage involves estimating the normal vectors of the point clouds, a critical pre-processing step preceding 3D reconstruction. To achieve this, the conventional principal component analysis (PCA) method [28] is employed on the point clouds. The neighboring points for each point within the point clouds are determined using the K-nearest neighbors (KNN) approach [29]. Ensuring that the normal vectors are consistently oriented outward, we employ the condition in Equation (5) to position them outside the approximated PCA plane.

(5) $$\bar{n}.\left(v_{p}-p\right)>0\;\;\;\;\forall p\;\in PC$$

In Equation (5). “$\bar{n}$” represents the approximate normal for point “p”, and “$v_{p}$” denotes the initial viewing angle associated with point “p”. The set of viewing angles corresponding to PC comprises the initial directions of the points, which are estimated from the external alignment of each phase in “$V_{c}$”. These initial directions are extracted from the voxel model.

To conduct a 3D statistical analysis of the generated 3D models, it is necessary to define the “QCF” statistical characteristic within 3D space. “QCF” quantifies the degree of twisting or obstruction present between the phases of the microstructure. Specifically, as the degree of twisting and obstruction within a microstructure increases, the “QCF” value also increases. Conversely, when there is less obstruction between phases, allowing for easier access between them, the “QCF” value decreases, approaching unity.

To establish a mathematical definition for the three-dimensional state of QCF, we make an assumption that the outcome of the three-dimensional reconstruction process for an $M_{PC}$ meshing model is presented as a three-dimensional triangular mesh. We also consider a predetermined size for the $M_{PC}$ model, implying that the vertices of the $M_{PC}$ triangular mesh are confined within a cube of specific dimensions (N × N × N. e.g., 2048) with the outer boundary of the mesh corresponding to these dimensions.

Next, we construct the 3D voxel model for $M_{PC}$ according to Equation (6). In this context, we further assume that $\left\{C_{i}\right\}$ represents the collection of individual components within $M_{PC}$.

(6) $${VXI}_{M}=\left\{\left[\left(i,j,k\right).(i+1,j+1,k+1)\right]|\exists r\left(i+\frac{1}{2},j+\frac{1}{2},k+\frac{1}{2}\right)inside\;C_{r}\right\}$$

The voxel model mentioned above constitutes a solid representation of the $M_{PC}$ model, effectively outlining the interior of the VX phases. Subsequently, we generate the grid graph model, denoted as $G_{m}:(V,E),$ based on the ${VXI}_{M}$ model as per Equation (7).

(7) $$V=\left\{\left(i,j,k\right)\;\;\;i,j,k\in \left[1,N\right]\right\}-{VXI}_{M}\;E=\left(x,y\right)\;\;\;x,y\in V\&y\in neighbor(x)$$

In Equation (4), $G_{m}:(V,E)$ comprises the vertices of a grid with dimensions N × N × N, with the exclusion of points associated with both the interior and periphery of the phases generated in ${VX}_{M}$. This graph exclusively encompasses the vertices situated within the voids of the microstructure. The edges within this graph are established between each voxel’s corresponding vertex, such as *x*, and all its neighbors, encompassing the vertices of neighboring voxels that share at least one edge with voxel *x*. To be more precise, this relationship is defined in Equation (8).

(8) $$\forall x=\left(i,j,k\right)\in V,\;neighbor\;\left(x\right)=\left\{y=\left(s.t.r\right)\in V:\left|x-y\right|\leq 2\right\}$$

The aforementioned graph represents a mesh model of the three-dimensional microstructure model $M_{PC}$, and it is worth noting that vertices and edges corresponding to the interior points of the phases have been omitted. Consequently, we introduce two distance definitions within $G_{m}:(V,E)$. For any pair of vertices x and y in $G_{m}:\left(V,E\right),$ SD represents the Euclidean distance between x and y, measured by the Euclidean metric, irrespective of the path traversed in the graph. On the other hand, SP distance denotes the shortest path distance between these two vertices, specifically within the graph.

A key aspect of this modeling is that the SP distance essentially equates to the geodesic length between two vertices within the microstructure. Given that no paths traverse through phases in the microstructure, the geodesic length is identical to SP distance. With these defined distances, we can formulate Equation (9) to describe the three-dimensional QCF.

(9) $${QCF\;(x,y)}_{3D}=\frac{SP(x,y)}{SD(x,y)}$$

In this stage, the three-dimensional microstructure reconstruction network is employed to recreate the external surfaces of the phase components, effectively integrating them into the three-dimensional microstructure. Within the optimization model outlined in this section, the QCF statistical index is leveraged to fine-tune the space allocation within the convolution network model, as previously detailed in the preceding section. As mentioned in the earlier section, the output of the multilayer perceptron model takes the form of a probability density function known as “$f_{\theta }$”, generated in the final stage of the eight-layer multilayer perceptron (MLP) [30] from the sigmoid output.

Before employing the function $f_{\theta }(P)$ as the output for constructing the phase procedure, we optimize this function using the sign agnostic method. To investigate deeper, we consider the following set for a transient microstructure plane:

(10) $${Pl}_{x0}=\left\{f_{\theta }\left(P\right)\forall p:<p-x_{0},n>=0\right\}$$

Here, “*n*” represents the central point on the plane, and “$x_{0}$” denotes the normal of the plane. Specifically, “$x_{0}$” is defined as $\left(\frac{N}{2},\frac{N}{2},z\right):z\in \left[1,N\right]$, and “*n*” is set to “$\bar{z}$”.

Next, we establish the probability density function $\bar{f}(P)$ for all points “p” situated within the plane defined as $<p-x_{0},n>=0$, following the formulation presented in Equation (11) for the initial model.

(11) $$\bar{f}\left(P\right)=\left\{\begin{array}{cc} \min\left(\max\left(\frac{3}{4},\frac{{QCF}_{l}\left(p\right)}{{QCF}_{ave.}\left(p\right)}\right),1\right) & p\in V\cup {VX}_{I}\\ \frac{1}{2} & p\notin V\cup {VX}_{I} \end{array}\right.$$

In the given equation, ${QCF}_{l}\left(p\right)$ represents the maximum value of QCF(p,y) for all “*y*” within the condition $\left|p-x\right|\leq l$, as defined in Relation (12).

(12) $${QCF}_{l}\left(p\right)=\max\left(QCF\left(p,y\right)\right)\forall y\in {VX}_{I}:\left|p-y\right|\leq l\;$$

Furthermore, for ${QCF}_{ave.}\left(p\right).$ we can refer to Relation (13).

(13) $${QCF}_{ave.}\left(p\right)=\max\left(QCF\left(x,y\right)\right)\forall x\in {VX}_{I}:\left|x-y\right|\leq l$$

In the above relations, the ${VX}_{I}$ model is the voxel model based on the point cloud and V vertices of the graph $G_{m}:(V,E)$. In the explanation of the above formula, the probability function $\bar{f}\left(P\right)$ is created in such a way that for the points that are on the edges of the phases in the voxel created from the model, it has a value between [3/4,1] and for the points that are on the edges of the phases have a small value equal to 1/2, and this means that a high probability is given for the edge points of the voxels to be seen on the top of the phases. Additionally, for the points that are not on the edge of the voxels, a small probability is given to be placed on surfaces the phases. The amount of probability attributed for a point is proportional to the amount of deviation around that point, in other words, the amount of access to that point from the radius l.

The probability assigned to a point is proportionate to the degree of distortion around that point, reflecting the accessibility within the radius l. In simpler terms, points located in the more intricate curves of a phase carry a higher probability, indicating their increased significance in optimization. Hence, employing this method results in a closer alignment of the number of bends and folds with those in the original model. Assuming H represents the standard binary mutual entropy for probability density functions, the optimization error function is defined for the application of sign agnostic in the form of Relation (14).

(14) $$l\left(f,\bar{f}\right)=\sum_{p\in {Pl}_{xo}\&f(p)>1/2}H(\bar{f}\left(p\right),f(p))$$

In this step, the sign-agnostic optimization method is applied for a limited number of repetitions to optimize the parameters of the model at each iteration. The initial filling of model parameters involves a pre-trained model, which has undergone numerous iterations on a substantial database. Subsequently, secondary optimization is performed to ensure the results align with the model’s curvatures. The final stage of the convolutional occupancy networks model involves creating a meshing model after training and determining the ultimate probability of occupancy. This is achieved through the multi-directional extraction method of iso surfaces. This method begins by identifying the occupied or potential points for point cloud input, followed by the application of the Marsh cube algorithm for meshing. To achieve this, the trained model of convolutional occupancy networks is transferred to the SCANNET database [31]. The model is then executed to ensure high-quality point clouds for effectively distinguishing model phases, utilizing the pre-trained model. Subsequent to this step, the point cloud structure undergoes transformation into a 3D mesh model.

## 3. Results and Discussion

**Figure 3:**
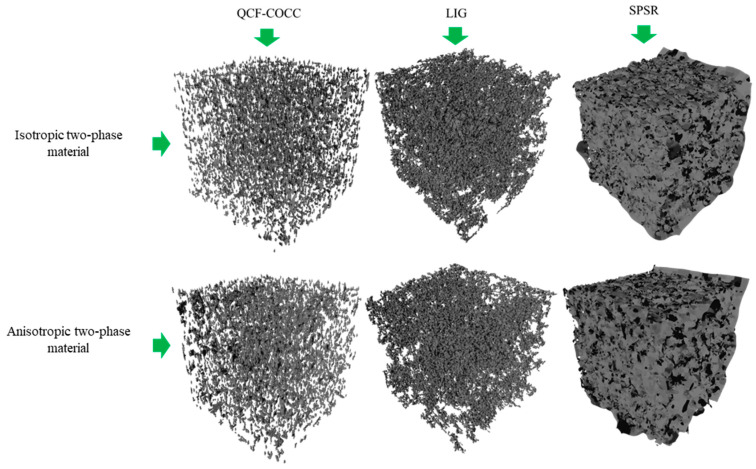
3D microstructure of the two-phase isotropic and anisotropic materials using QCF-COCC. LIG and SPSR.

Using two-dimensional images of the microstructure, we successfully reconstructed the 3D microstructures of materials using our model (QCF-COCC) and compared it with other models, including those employing local implicit grid (LIG) representations for 3D scenes and Screened Poisson Surface Reconstruction (SPSR). The reconstructions were successful for isotropic materials as well as anisotropic materials with two and three phases. Figure 3 depicts the results for the isotropic and anisotropic two-phase materials. It is evident from the images that our model achieves superior microstructure details, particularly in the inner regions of the considered volume. Furthermore, our method precisely distinguishes the phases of the materials without any phase separation. The reconstructions appear to have smooth surfaces and defined edges, suggesting that QCF is effective at capturing the continuous phase of the materials while potentially smoothing over some finer details. Although the LIG model can also reconstruct the microstructure of the two-phase materials, it yields lower details, especially in the inner regions. The reconstructions appear slightly less smooth and more pixelated than QCF, which could indicate that LIG retains more of the local geometric detail but may also introduce some discretization artifacts due to the grid representation. In contrast, the SPSR model struggles with the reconstruction of the 3D microstructure of the two-phase isotropic and anisotropic materials, as evidenced by significant difficulties in distinguishing between the two phases. SPSR captures the fine structural details of the materials well, but this also comes with increased noise, which can be seen as a speckled appearance on the surface.

**Figure 4:**
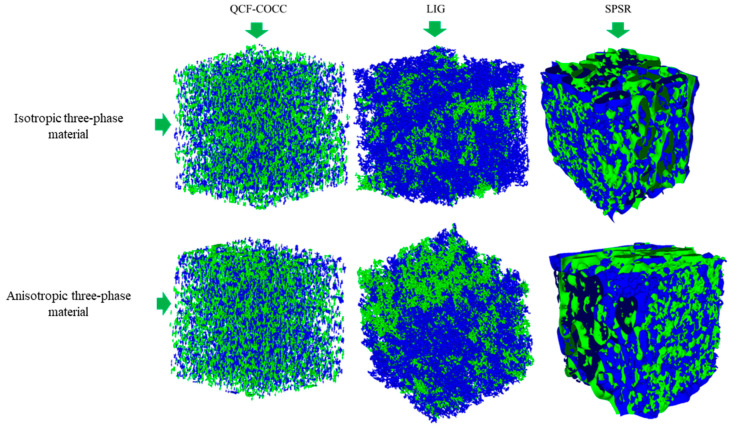
3D microstructure of the three-phase isotropic and anisotropic materials using QCF-COCC. LIG and SPSR.

To further demonstrate the capabilities of the developed model in 3D reconstruction of microstructures, the results for isotropic and anisotropic three-phase materials are presented alongside those of other models in Figure 4. Two distinct colors are used to illustrate the phases of the materials. The images in Figure 4 clearly show that our model can precisely reconstruct the microstructure of the three-phase materials. The phases are clearly defined in the 3D structure. Each phase precisely connected without any separation during the reconstruction. However, upon examining the images of the microstructures developed by other models, it becomes evident that these 3D reconstruction models are less capable of precise detail rendering. It can be observed that they typically reconstruct only a basic layer, overlooking finer details.

Generally, we can conclude that reconstructions demonstrating cleaner and more distinct phase boundaries typically signify an enhanced performance of QCF-COCC in defining phase connectivity. This capability is critical for accurate material characterization. The model developed herein consistently exhibited precise demarcation between phases, without blending or ambiguity at their interfaces, thereby suggesting superior phase definition. Furthermore, our approach effectively maintains the continuity of individual phases throughout the structure, distinctly outperforming the LIG technique in this respect. Additionally, the QCF-COCC method has demonstrated remarkable proficiency in revealing intricate internal structures, such as textural details and complex phase interfaces. significantly surpassing the capabilities of the SPSR method. Collectively, these findings underscore the robustness of our model in accurately representing the complex internal composition of materials, thereby facilitating a more precise analysis of materials’ properties.

**Figure 5:**
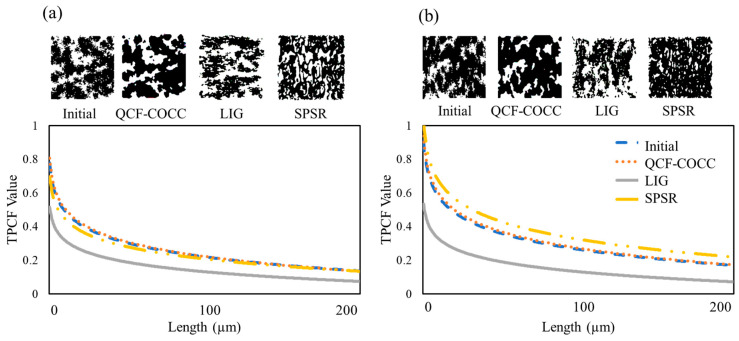
Two-point correlation (TPCF) values for the initial images and 2D cut section of the microstructure from different 3D microstructure of the two-phase materials: (**a**) isotropic and (**b**) anisotropic.

For statistical investigation of the developed microstructures and verification of the models, the two-point correlation function (TPCF) was derived for the cut section of the microstructure and compared with the input images. The results of TPCF for the two-phase isotropic and anisotropic materials are depicted in the diagrams of Figure 5.

For the isotropic materials, as shown in Figure 5a, the TPCF values for the 2D cut section of the microstructures derived from the QCF-COCC and SPSR methods are relatively similar to the initial input. However, the results for the 2D cut section of the LIG method differ from those of other methods and the initial input.

For the anisotropic materials, as shown in Figure 5b, only the results from the QCF-COCC method are similar to the initial input, while other methods show deviations from the initial input results. This deviation is more pronounced for the LIG method compared to the SPSR technique.

These results demonstrate the capability of our method in the precise reconstruction of the microstructure of two-phase isotropic and anisotropic materials.

**Figure 6:**
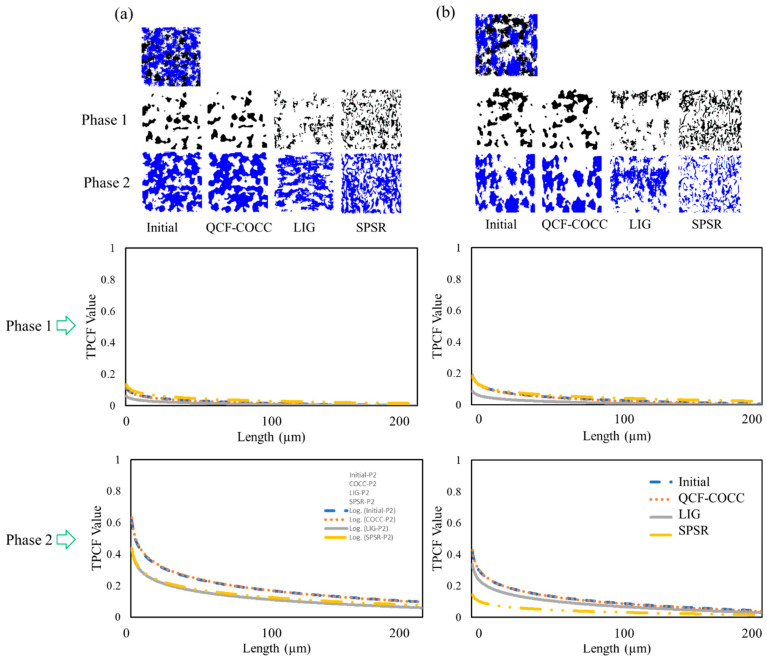
Two-point correlation (TPCF) values for the initial images and 2D cut section of the microstructure from different 3D microstructure of the three-phase materials: (**a**) isotropic and (**b**) anisotropic.

The TPCF results of the three-phase isotropic and anisotropic materials are depicted in the diagrams of Figure 6. Since the materials have three phases, the TPCF values were calculated separately for each phase. As shown in the diagrams, the TPCF values for our model closely match the initial input values. However, for other models the results for all phases deviate from the initial values and from those of our model. This further demonstrates the capability of our model in the 3D reconstruction of the microstructure of three-phase materials.

**Figure 7:**
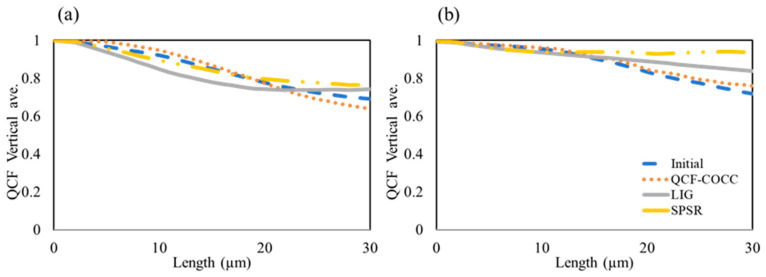
QCF values for the initial images and 2D cut section of the microstructure from different 3D microstructures of the two-phase materials: (**a**) isotropic and (**b**) anisotropic.

**Figure 8:**
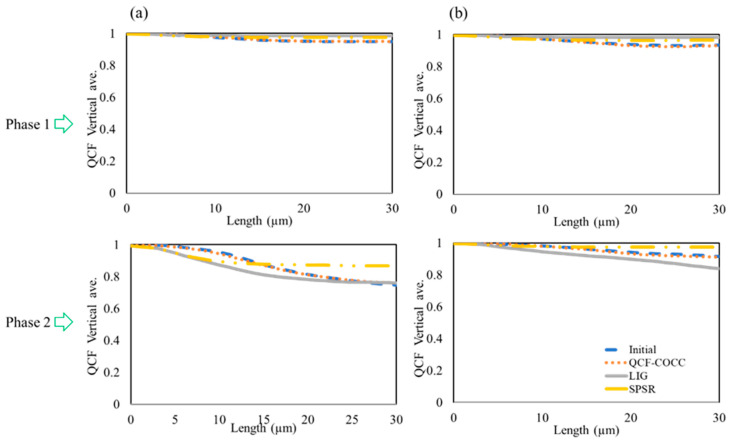
QCF values for the initial images and 2D cut section of the microstructure from different 3D microstructures of the three-phase materials: (**a**) isotropic and (**b**) anisotropic.

For further statistical analysis of the different models, QCF values were derived for the same 2D section of the structure. The results for the two-phase and three-phase materials are presented in Figure 7 and Figure 8, respectively. Figure 7 illustrates that for the two-phase isotropic and anisotropic materials the QCF trends in both isotropic and anisotropic materials for the initial image and the section derived from QCF-COCC methods are consistent. However, other methods exhibit a difference in this trend, with QCF values deviating more from the initial images. The diagrams for the three-phase materials show a similar trend to the two-phase materials, further validating the results and precision of our model in the 3D reconstruction of the microstructure of different materials. It is worth mentioning the reasons for selecting the LIG and SPSR methods for comparison with our methods. SPSR is known for its ability to reconstruct smooth 3D surfaces, which is particularly useful in computer graphics. However, our study focuses on the reconstruction of complex internal microstructures of materials, which often include intricate and detailed features. By comparing QCF-COCC with SPSR, we illustrate the limitations of traditional methods like SPSR when applied to material microstructures, demonstrating QCF-COCC’s superior capability in capturing fine internal structures. Similarly, the LIG method, which excels in handling noisy data through localized implicit surfaces, was chosen to show how QCF-COCC maintains detailed phase connectivity and structural fidelity in heterogeneous materials. These comparisons highlight QCF-COCC’s robustness and versatility across different 3D reconstruction challenges, underscoring its generalizability and effectiveness in accurately reconstructing both smooth and complex microstructures.

## 4. Conclusions

In this study, we have advanced the field of 3D reconstruction by developing an optimized convolutional occupancy network that incorporates a Quality of Connection Functions (QCF) model. This innovative approach has been specifically tailored for the 3D reconstruction of various materials, encompassing both two-phase and three-phase systems, as well as isotropic and anisotropic materials. The primary contribution of this paper lies in the successful integration of QCF, which significantly enhances the accuracy and detail in the 3D reconstructions of these complex material systems. This development not only improves the fidelity of material modeling but also broadens the potential applications in materials science and engineering. The main contributions of this paper are as follows:

1. It was demonstrated that the QCF-COCC model excels in reconstructing the 3D microstructure of both isotropic and anisotropic materials with two and three phases. This model notably outperforms other methods such as Screened Poisson Surface Reconstruction (SPSR) and Local Implicit Grid (LIG) representations, particularly in capturing detailed features within the microstructures’ inner regions.
2. In terms of phase accuracy and detail, the QCF-COCC model accurately distinguishes and connects different phases in reconstructed 3D structures without any phase separation. This represents a significant advantage over other examined models, which frequently struggle with phase distinction and detailed reconstruction.
3. Visual comparisons and statistical analyses confirm the superior performance of the QCF-COCC model. This model not only replicates the intricate geometries of various materials structures with greater accuracy but also maintains high fidelity to the original 2D images used as inputs.
4. The QCF-COCC model significantly advances 3D microstructure reconstruction, showing consistent QCF trends across various materials. The model’s superior phase connectivity and structural fidelity, validated by close alignment of QCF and TPCF values of cut-sections of reconstructed microstructure with initial images, underscore its accuracy and reliability.

The QCF-COCC model demonstrates significant applicability in fields that require precise structure–property linkages, such as finite element analysis. Its capability to produce detailed and accurate 3D reconstructions from limited 2D images represents a substantial advancement over traditional stochastic and practical methods, providing a faster and more cost-effective alternative.

## Data Availability

The original contributions presented in the study are included in the article, further inquiries can be directed to the corresponding authors.

## References

[1] Sebdani M.M., Baniassadi M., Jamali J., Ahadiparast M., Abrinia K., Safdari M. (2015). Designing an optimal 3D microstructure for three-phase solid oxide fuel cell anodes with maximal active triple phase boundary length (TPBL). Int. J. Hydrogen Energy.

[2] Brahme A., Alvi M., Saylor D., Fridy J., Rollett A. (2006). 3D reconstruction of microstructure in a commercial purity aluminum. Scr. Mater..

[3] Uchic M.D., Holzer L., Inkson B.J., Principe E.L., Munroe P. (2007). Three-dimensional microstructural characterization using focused ion beam tomography. MRS Bull..

[4] Echlin M.P., Burnett T.L., Polonsky A.T., Pollock T.M., Withers P.J. (2020). Serial sectioning in the SEM for three dimensional materials science. Curr. Opin. Solid State Mater. Sci..

[5] Li X., Duan L., Zhou S., Liu X., Yao Z., Yan Z. (2024). Freeze-Casting of Alumina and Permeability Analysis Based on a 3D Microstructure Reconstructed Using Generative Adversarial Networks. Materials.

[6] Mura F., Cognigni F., Ferroni M., Morandi V., Rossi M. (2023). Advances in Focused Ion Beam Tomography for Three-Dimensional Characterization in Materials Science. Materials.

[7] Seyed Mahmoud S.M.A., Faraji G., Baghani M., Hashemi M.S., Sheidaei A., Baniassadi M. (2023). Design of Refractory Alloys for Desired Thermal Conductivity via AI-Assisted In-Silico Microstructure Realization. Materials.

[8] Jing H., Dan H., Shan H., Liu X. (2023). Investigation on Three-Dimensional Void Mesostructures and Geometries in Porous Asphalt Mixture Based on Computed Tomography (CT) Images and Avizo. Materials.

[9] Groeber M.A., Haley B., Uchic M.D., Dimiduk D.M., Ghosh S. (2006). 3D reconstruction and characterization of polycrystalline microstructures using a FIB–SEM system. Mater. Charact..

[10] Xu H., Bae C. (2019). Stochastic 3D microstructure reconstruction and mechanical modeling of anisotropic battery separators. J. Power Sources.

[11] Landis E.N., Keane D.T. (2010). X-ray microtomography. Mater. Charact..

[12] Brilakis I., Fathi H., Rashidi A. (2011). Progressive 3D reconstruction of infrastructure with videogrammetry. Autom. Constr..

[13] Politis M., Kikkinides E., Kainourgiakis M., Stubos A. (2008). A hybrid process-based and stochastic reconstruction method of porous media. Microporous Mesoporous Mater..

[14] Zhang W., Song L., Li J. (2019). Efficient 3D reconstruction of random heterogeneous media via random process theory and stochastic reconstruction procedure. Comput. Methods Appl. Mech. Eng..

[15] Wang G., Ye J.C., De Man B. (2020). Deep learning for tomographic image reconstruction. Nat. Mach. Intell..

[16] Reader A.J., Corda G., Mehranian A., da Costa-Luis C., Ellis S., Schnabel J.A. (2020). Deep learning for PET image reconstruction. IEEE Trans. Radiat. Plasma Med. Sci..

[17] Zhang H.-M., Dong B. (2020). A review on deep learning in medical image reconstruction. J. Oper. Res. Soc. China.

[18] Yamada H., Liu C., Wu S., Koyama Y., Ju S., Shiomi J., Morikawa J., Yoshida R. (2019). Predicting materials properties with little data using shotgun transfer learning. ACS Cent. Sci..

[19] Zhang Y., An M. (2017). Deep learning-and transfer learning-based super resolution reconstruction from single medical image. J. Healthc. Eng..

[20] Li X., Zhang Y., Zhao H., Burkhart C., Brinson L.C., Chen W. (2018). A transfer learning approach for microstructure reconstruction and structure-property predictions. Sci. Rep..

[21] Bostanabad R. (2020). Reconstruction of 3D microstructures from 2D images via transfer learning. Comput. Aided Des..

[22] Xu Y., Weng H., Ju X., Ruan H., Chen J., Nan C., Guo J., Liang L. (2021). A method for predicting mechanical properties of composite microstructure with reduced dataset based on transfer learning. Compos. Struct..

[23] Bagherian A., Famouri S., Baghani M., George D., Sheidaei A., Baniassadi M. (2022). A new statistical descriptor for the physical characterization and 3D reconstruction of heterogeneous materials. Transp. Porous Media.

[24] Gerstner T., Pajarola R. Topology preserving and controlled topology simplifying multiresolution isosurface extraction. Proceedings of the Visualization 2000. VIS 2000 (Cat. No.00CH37145).

[25] Kazhdan M., Hoppe H. (2013). Screened poisson surface reconstruction. ACM Trans. Graph..

[26] Jiang C., Sud A., Makadia A., Huang J., Nießner M., Funkhouser T. Local implicit grid representations for 3d scenes. Proceedings of the IEEE/CVF Conference on Computer Vision and Pattern Recognition.

[27] Baniassadi M., Garmestani H., Li D., Ahzi S., Khaleel M., Sun X. (2011). Three-phase solid oxide fuel cell anode microstructure realization using two-point correlation functions. Acta Mater..

[28] Kurita T. (2019). Principal component analysis (PCA). Computer Vision: A Reference Guide.

[29] Peterson L.E. (2009). K-nearest neighbor. Scholarpedia.

[30] Safar A.A., Salih D.M., Murshid A.M. (2023). Pattern recognition using the multi-layer perceptron (MLP) for medical disease: A survey. Int. J. Nonlinear Anal. Appl..

[31] Dȩbska B. (1992). SCANNET: A spectroscopy database. Anal. Chim. Acta.

